# Retraction of ‘known sequence features can explain half of all human gene ends’

**DOI:** 10.1093/nargab/lqad028

**Published:** 2023-04-05

*NAR Genomics and Bioinformatics*, Volume 3, Issue 2, June 2021, lqad031, https://doi.org/10.1093/nargab/lqab042

In December 2022, the Authors alerted the Editors that the Cryptic_dataset.bed file originally posted on the project web site (https://hugheslab.ccbr.utoronto.ca/supplementary-data/HumanGeneEnds/) , is erroneous. This file gave the locations and scores of all the cryptic Cleavage and Polyadenylation (CPA) sites discovered by the ‘baseline’ Random Forests classifier, and it was used in later parts of the paper, mainly Figure 6.

To rebuild the file, all the analyses in the article were repeated, confirming most of the results and conclusion of the article. However, Figure 6 and the analyses supporting the figure were affected, as detailed below.

- Analyses with the correct file did not show the dramatic improvement in real vs. cryptic CPA site classification that was originally claimed.
- The real vs. cryptic model also retained many fewer RBP motifs than originally claimed – instead, it retained one or two motifs, depending on random initialization, each resembling the known PAS (Polyadenylation site) .
- Shuffled motifs performed no better than real motifs (another result in Figure 6) , presumably because the PAS is mostly a homopolymer (consensus AAUAAA) , so shuffling it (and related motifs) produces a related homopolymer.

The corrected Figure 6 therefore reaches a very different biological conclusion from the original paper. The original paper speculated that many factors make small contributions to CPA site definition, but this new result instead suggests only that the current models of the PAS sequence will benefit from tweaking.

Given the circumstances, the Authors are retracting the original version of the article and replacing it with a new version, which has been reviewed and accepted by the editors. The new version has been republished as https://doi.org/10.1093/nargab/lqad031. The .bed file has been replaced with the rebuilt version on the project web site.

